# Electroacupuncture at the Zusanli (ST-36) Acupoint Induces a Hypoglycemic Effect by Stimulating the Cholinergic Nerve in a Rat Model of Streptozotocine-Induced Insulin-Dependent Diabetes Mellitus

**DOI:** 10.1093/ecam/neq068

**Published:** 2011-02-14

**Authors:** Yu-Chen Lee, Te-Mao Li, Chung-Yuh Tzeng, Ying-I Chen, Wai-Jane Ho, Jaung-Geng Lin, Shih-Liang Chang

**Affiliations:** ^1^Department of Acupuncture, China Medical University Hospital, Taiwan; ^2^School of Chinese Medicine, China Medical University, Taichung City, Taiwan; ^3^Department of Orthopedics, Taichung Veterans General Hospital, Taichung City, Taiwan; ^4^Department of Medicinal Botanicals and Health Care, Da-Yeh University, Chunghwa County, Taiwan

## Abstract

Animal studies have shown that electroacupuncture (EA) at Zusanli (ST-36) and Zhongwan (CV-12) acupoints reduces plasma glucose concentrations in rats with type II diabetes. However, whether EA reduces plasma glucose levels in type I diabetes is still unknown. In this study, we explore the various non-insulin-dependent pathways involved in EA-induced lowering of plasma glucose. Streptozotocin (STZ) (60 mg kg^−1^, i.v.) was administered via the femoral vein to induce insulin-dependent diabetes in non-adrenalectomized and in adrenalectomomized rats. EA (15 Hz) was applied for 30 min to bilateral ST-36 acupoints after administration of Atropine (0.1 mg kg^−1^ i.p.), Eserine (0.01 mg kg^−1^ i.p.), or Hemicholinium-3 (5 *μ*g kg^−1^ i.p.) in non-adrenalectomized rats. Rats administered acetylcholine (0.01 mg kg^−1^ i.v.) did not undergo EA. Adrenalectomized rats underwent EA at bilateral ST-36 acupoints without further treatment. Blood samples were drawn from all rats before and after EA to measure changes in plasma glucose levels. Expression of insulin signaling proteins (IRS1, AKT2) in atropine-exposed rats before and after EA was measured by western blot. Atropine and hemicholinium-3 completely blocked the plasma glucose lowering effects of EA, whereas eserine led to a significant hypoglycemic response. In addition, plasma glucose levels after administration of acetylcholine were significantly lower than the fasting glucose levels. In STZ-adrenalectomized rats, EA did not induce a hypoglycemic response. EA stimulated the expression of IRS1 and AKT2 and atropine treatment blocked the EA-induced expression of those insulin signaling proteins. Taken together, EA at the ST-36 acupoint reduces plasma glucose concentrations by stimulating the cholinergic nerves.

## 1. Introduction

Diabetes is an important public health issue worldwide. Traditional Chinese medicine treatments for diabetes include electroacupuncture (EA), syndrome differentiation and Chinese massage (Tui na) [1–4]. EA and Tui na not only decrease plasma glucose levels, but also stimulate the cardiovascular system, the neuroendocrine system and the digestive system to either directly or indirectly regulate plasma glucose [5–7].

Methods for controlling plasma glucose levels in patients with diabetes mellitus (DM) include diet control, exercise and medication. While medical treatment is convenient and effective, certain treatments can lead to serious side effects [8, 9]. Patients with poorly controlled non-insulin-dependent, type II DM who frequently use oral hypoglycemic agents often present with unstable plasma glucose levels. In addition, insulin resistance due to long-term insulin usage is common among patients with insulin-dependent type I DM. Researchers have, therefore, searched for mild or complementary treatment methods with no side effects to increase insulin sensitivity [2, 5, 6, 10]. Studies on EA treatment of diabetes in animal models have shown that EA leads to a reduction in plasma glucose levels by promoting insulin production and improves insulin sensitivity by inducing secretion of endogenous *β*-endorphin. Studies have also shown that stimulating the CV-12, CV-4 and ST-36 acupoints with a specific frequency on both sides of rats significantly reduces their plasma glucose levels [5, 11]. Stimulation of acupoints results in significantly greater plasma glucose lowering effects than stimulation of adjacent non-acupuncture points [5]. Researches exploring this mechanism discovered that the insulin secretion encouraged by EA originates from endorphin stimulation, and that sufficient amounts of the opioid receptor binding blocker naloxone (1 mg kg^−1^, i.v.) inhibits the EA-induced plasma glucose lowering effect [5]. Lin et al. showed that 15 Hz EA at the CV-12 acupoint in adrenalectomized rats resulted in reduced plasma glucose levels [12]. A relative study showed that a stimulation frequency of 2 Hz have a minor plasma glucose lowering effect [13]. Lin et al. concluded that multiple sources of endogenous opioid peptide participate in the hypoglycemic effect of EA (15 Hz) stimulation at the CV-12 acupoint [12]. Researchers compared the plasma glucose lowering effect of low frequency (2 Hz) EA stimulation at the ST-36 acupoint with that at the CV-12 acupoint and found that the ST-36 acupoint was more effective in reducing plasma glucose levels. Studies have repeatedly observed that effect in adrenalectomized rats [14]. It has been shown that the hypoglycemic effect of low frequency (2 Hz) EA stimulation at the ST-36 acupoint in adrenalectomized rats was not attenuated by naloxone treatment [15]. This finding implies that the glucose lowering effects of 2 Hz EA applied to ST-36 acupoints do not involve the endogenous opioid peptide mechanism only. Whether or not the parasympathetic nervous system participates in the plasma glucose lowering effect of EA is an interesting question. Acetylcholine (Ach) affects and relaxes vascular smooth muscles and lowers blood pressure. This occurs mainly through binding to the M3 receptor, which results in an increase in intracellular Ca^2+^ levels, which in turn leads to eNOS activation and NO production. The parasympathetic nerve blocker atropine blocks muscarinic receptors and directly competes with Ach for the M3 receptor. The nerve blocker hemicholinium (HC-3) represses the choline re-uptake channel of cholinergic nerves to directly repress Ach production instead of acting upon the nerve transmission system [16]. The Chinese scholar Zhao regulated the parasympathetic nervous system and eNOS expression using sheep red blood cell (SRBC) to induce an immune response and HC-3 blocker to test the role of the parasympathetic nervous system in immune system regulation induced by EA. Zhao used the lymphocyte transformation test (LTT) and measured interlukin-2 (IL-2) activity to evaluate whether the EA-induced immune system regulatory effects are due to the release of Ach by parasympathetic nerves [17]. Hsieh et al. tested the effects of low frequency (2 Hz) and high frequency (100 Hz) EA stimulation at the ST-36 acupoint on heart rate and skin temperature in humans and found that high-frequency EA stimulation led to a reduction in heart rate by activating parasympathetic nerves [18]. Previous studies have shown that EA has plasma glucose lowering effects in non-insulin-dependent types; however, the plasma glucose lowering effects and the mechanisms by which EA induces a hypoglycemic effect in insulin-dependent types is not well understood.

Although acupuncture is not suitable as a long-term treatment for diabetes, it may be suitable as a short-term intervention in patients with unstable plasma glucose control [6, 19, 20]. Previously, we explored various insulin-dependent pathways through which EA induces hypoglycemia [5]. In this study, we explore the various non-insulin-dependent pathways involved in EA-induced lowering of plasma glucose.

## 2. Methods

### 2.1. Animal Models

Normal male Wistar Rats weighing *∼*250–350 g and aged 8–10 weeks were purchased from the BioLASCO animal center. After a 1-week adaptation period, animals were randomly divided into two groups. In Group I, insulin-dependent diabetes was induced by administration of streptozotocin (STZ) (60 mg kg^−1^, i.v.) via the femoral vein on the third day of a 3-day fasting period as described previously [11, 21]. In Group II, rats underwent bilateral adrenalectomy. After a 3-day recovery period, insulin-dependent diabetes was induced by administration of STZ (60 mg kg^−1^, i.v.) via the femoral vein as previously described [12, 13]. Animals were housed in plexiglass cages at a constant room temperature of 22 ± 2°C with a relative humidity of 65 ± 5%. Rats were fed standard rat chow and were given free access to water. Animals were treated in accordance with the National Institute of Health (NIH) Guide for the Care and Use of Laboratory Animals, and the study protocol was approved by the ethics committee of the China Medical University, Taichung, Taiwan.

### 2.2. EA

Acupoints were located according to body-length measurement as described elsewhere [22]. The ST-36 acupoint was located on the anterior tibia muscle approximately upper 1/6 to the length of lower leg below the knee. Bilateral ST-36 acupoints were punctured in a vertical and deep manner with 1.27-cm, 32-gauge acupuncture needles. After a 5-min needling period, EA was performed for 30 min at a frequency of 15 Hz and an amplitude of 10 mA using a HANS LY257 acupoint and nerve stimulator (Healthtronics, Singapore).

### 2.3. Plasma Glucose Assay

Animals were anesthetized using pentobarbital (40 mg kg^−1^ i.p). Approximately 0.3–0.5 mL of blood was obtained from a femoral vein using a 1-mL syringe containing heparin. The collected blood was introduced into eppendorff tubes, lightly shaken and then stored on ice. Following centrifugation at 21 880 × g for 5 min, a Glucose UV reagent (Raichem, USA) was added to test the amount of biological index glucose contained in the serum. The content was measured using a fully automatic biochemical analyzer (Roche COBAS-MIRA-PLUS, USA).

### 2.4. Experimental Protocol

#### 2.4.1. Electrical Acupuncture Hypoglycemic Experiment

STZ-induced diabetic rats (*n* = 16) were randomly divided into the EA group (*N* = 8) or the control group (*N* = 8). Animals in the EA group were anesthetized and then subjected to EA for 30 min as described in the Methods section. Rats in the control group were anesthetized but did not undergo EA. Blood was extracted for glucose testing prior to the experiment and 30 min after the experiment as previously described.

#### 2.4.2. Examination of EA Impact on Glucose Mechanisms

*Atropine Experiment*: Atropine 0.1 mg kg^−1^ was injected into the abdomens of 12 STZ-induced diabetic rats 30 min prior to the experiment. The 12 rats were randomly and equally divided into the EA group or the control group. Blood was extracted for glucose testing prior to the experiment and 30 min after the experiment.

*Eserine Experiment*: Eserine 0.01 mg kg^−1^ was injected into the abdomens of 12 STZ-induced diabetic rats 30 min prior to the experiment. The rats were then randomly divided into the EA group (*N* = 6) or the control group (*N* = 6). Blood was extracted for glucose testing immediately before and 30 min after the experiment.

*Hemicholinium (HC-3) Experiment*: HC-3 (5 *μ*g kg^−1^) was injected into the abdomens of 12 STZ-induced diabetic rats 30 min prior to the experiment. The 12 rats were then randomly and equally divided into the EA group or the control group. Blood was extracted for glucose testing 30 min before and 30 min after the experiment.

*Ach Experiment*: This experiment was performed on six STZ-induced diabetic rats. Blood was extracted from anesthetized animals 30 min prior to the administration of Ach 0.01 mg kg^−1^ i.v. EA was not performed. Blood was extracted for glucose testing 0, 30 and 60 min after the beginning of the experiment.

*EA in Adrenalectomized Rats*: A total of 12 STZ-induced diabetic rats that had undergone adrenalectomy were randomly divided into the EA group (*N* = 6) or the control group without EA (*N* = 6). Blood was extracted for glucose testing 30 min before and 30 min after the experiment.

#### 2.4.3. Western Blot Assay

Fasting STZ rats (*n* = 12) were randomly divided into EA or non-EA groups. The other fasting STZ rats (*n* = 12) were randomly divided into the EA or non-EA group 30 min after treatment with atropine 0.1 mg kg^−1^, i.p. At the end of treatment (30 min) in each group, portions of the gastrocnemius muscles were taken as samples for analysis of insulin signaling proteins (IRS1, AKT2). The samples were homogenized in buffer solution before centrifugation at 16 440 × g. The obtained supernatant was used to estimate the amount of protein using an assay kit (Bio-Rad Laboratories, CA, USA). The supernatant (protein) was added to a 4× loading dye and boiled for 15 min at 95°C for denaturing. This process produced a separating (8%) and stacking gel. Then, protein (90 *μ*g mL^−1^) in the buffer solution was loaded into each well for electrophoresis. Proteins were electrophoretically transferred to polyvinylidene difluoride membranes at 4°C. The membranes were then blocked with 5% nonfat dry milk in phosphate buffered saline (PBS) for 1 h at room temperature and incubated with specific primary antibodies (Santa Cruz Biotechnology, Inc., CA, USA). After washing the membranes in a buffer containing 0.1% Tween 20 in 1 × PBS, blots were incubated with a horseradish peroxidase-linked specific second antibody (Santa Cruz Biotechnology, Inc.) followed by enhanced chemiluminescence detection using ECL reagent plus (PerkinElmer Life Sciences, Inc., USA). Band intensities were quantified by densitometry to observe the target proteins. *β*-actin served as a loading control.

### 2.5. Statistical Analysis

The experimental results for each group are expressed in means ± SEM; *t*-test and ANOVA statistical analyses were performed. Statistically significant differences were set at *P* < .05.

## 3. Results

### 3.1. Hypoglycemic Effect

**Figure 1:**
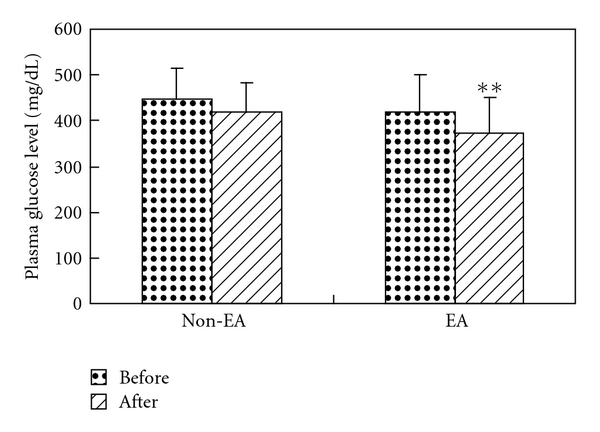
Hypoglycemic effects of ST-36 EA in STZ rats: EA represents fasting for 12 h and EA treatment for 30 min; non-EA represents the non-EA control group; comparison of plasma glucose levels was performed by self-pair *t*-test, **P < .01.

EA (15 Hz) was applied to bilateral ST-36 acupoints in eight STZ-induced diabetic rats. As seen in Figure 1, there was a significant decrease in mean plasma glucose levels before EA (418 ± 82 mg dL^−1^) and 30 min after EA (372 ± 77 mg dL^−1^) (*n* = 8, *P* < .01). There were no significant differences in mean plasma glucose levels in the control (non-EA) group (range 447 ± 69 to 420 ± 61 mg dL^−1^; *P* > .05).

### 3.2. The Influence of Atropine, HC-3 and Eserine

Atropine (0.1 mg kg^−1^ i.p.) and HC-3 (5 *μ*g kg^−1^ i.p.) blocked the hypoglycemic effects of 15 Hz EA performed on the ST-36 acupoint. Thirty minutes following treatment, mean plasma glucose levels were 413 ± 29 mg dL^−1^ in the atropine group and 510 ± 109 mg dL^−1^ in the HC-3 group. After EA stimulation, mean plasma glucose levels had decreased by 1 ± 4% to 408 ± 32 mg dL^−1^ in the atropine group and by 3 ± 7% to 494 ± 106 mg dL^−1^ in the HC-3 group. There were no significant differences before and after EA treatment in either group. In addition, there were no significant differences in mean plasma glucose levels after injection with atropine or HC-3 in the control group (*P* > .05) (Table 1). Conversely, 15 Hz EA stimulation in STZ-induced diabetic rats treated with eserine (0.01 mg kg^−1^ i.p.) led to a reduction in mean plasma glucose levels. Mean glucose levels were 406 ± 55 mg dL^−1^ following eserine treatment and 331 ± 57 mg dL^−1^ 30 min after EA treatment, representing a decrease of 19 ± 6% (*n* = 6). The difference in mean values before and after EA was significant (*P* < .01).

### 3.3. The Effect of Ach on STZ Rats

Plasma glucose values were measured in STZ-induced diabetic rats before and after administration of Ach (0.01 mg kg^−1^ i.v.). The mean fasting plasma glucose level was 491 ± 47 mg dL^−1^ before treatment and 408 ± 71 mg dL^−1^ after 60 min treatment with Ach, representing a decrease of 18 ± 10% (*n* = 7). The difference between plasma glucose levels before and after treatment was significant (*P* < .05).

### 3.4. The Impact of Adrenalectomy on EA

EA (15 Hz) was applied to both ST-36 acupoints in STZ-adrenalectomized rats. Plasma glucose levels were initially 507 ± 39 mg dL^−1^, while plasma glucose levels following EA were 494 ± 63 mg dl^−1^, representing a decrease of 3 ± 6% (*n* = 6). The difference in mean plasma glucose levels before and after EA was not significant (*P* > .05). In addition, there were no significant differences in mean hypoglycemic activity between STZ-adrenalectomized rats with EA and without EA stimulation (Table 2, *P* > .05).

### 3.5. EA-Induced Expression of Insulin Signal Proteins

**Figure 2:**
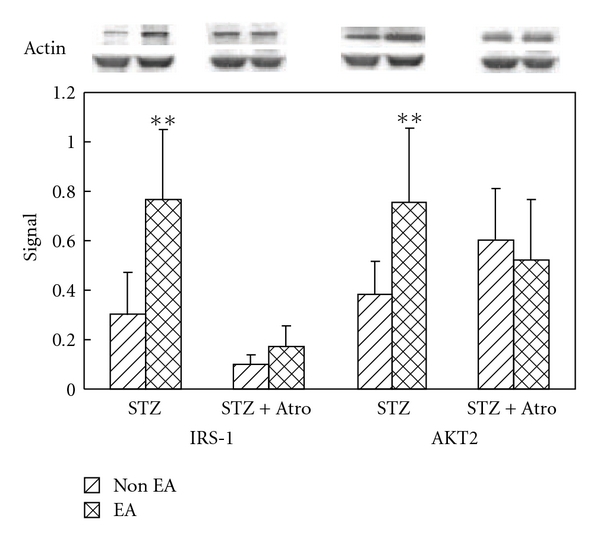
Effect of EA and atropine on the expression of muscular insulin signaling proteins. All signaling protein values were calculated as a ratio of signal protein to actin. The ratios were compared within the groups. Signal: signal protein/actin ratio, STZ group: the STZ-induced diabetic rats were treated with normal saline as a control, STZ + Atro group: the STZ-induced diabetic rats were treated with atropine 0.1 mg kg^−1^ for 30 min before EA and non-EA. The Student's *t*-test was used to compare the means between the EA and non-EA groups, **P < .01 was considered statistically significant.

The levels of IRS1 and AKT2 protein expression were significantly lower in the non-EA group than in the EA group. There were no significant differences in IRS1 and AKT2 expression between the EA and non-EA groups that had been administered atropine (Figure 2).

## 4. Discussion

We found that EA stimulation at the ST-36 acupoint for 30 min resulted in a significant reduction in plasma glucose levels in STZ-induced diabetic rats. These results are consistent with those reported by Lee [23] and Chang [11] that indicated acupuncture and EA performed on ST-36 acupoints lowered plasma glucose levels in type I diabetic animal models. We did not evaluate the effect of EA at non-acupoints in the study because a previous study showed that EA stimulation of the zhongwan acupoint resulted in a more significant plasma glucose lowering effect than EA stimulation at non-acupoints [5]. Thus, we concluded that the hypoglycemic effect of EA stimulation at acupoint is not only due to activation of 5′-AMP-activated protein kinase [24] caused by the contraction of electrically stimulated muscle, but also relative with stimulating different sites of acupoint that may more effective than the site of non-acupoint according to the meridian theory of traditional Chinese medicine.

**Figure 3:**
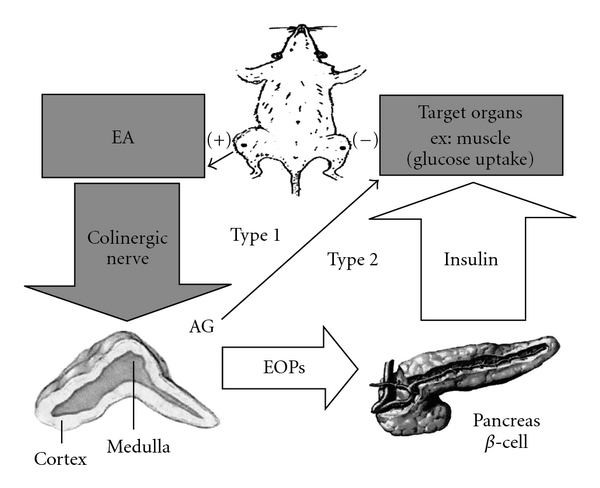
Hypoglycemic effect of EA in different types of DM. The gray block and arrow indicate the main pathway of the present study; the open arrows summarize the pathway suggested from previous study in non-insulin-dependent DM (type 2) [5]; the solid thin arrow indicates the direction of stimulation. Type 1: insulin-dependent DM; type 2: non-insulin-dependent DM; AG: adrenal gland; EOPs: endogenous opioid peptides; (+) (–): bilateral zusanli acupoints (ST-36) connected to the positive and negative pole of the EA apparatus.

This study examined whether the hypoglycemic effect of 15 Hz EA at the ST-36 acupoints in STZ-induced diabetic rats is mediated by the cholinergic nerve. A diagram of the hypothetical mechanism is depicted in Figure 3. We first experimented with the cholinergic nerve-blocking agent atropine. Our findings show that atropine (0.1 mg kg^−1^ i.p.) completely blocks the hypoglycemic effect of 15 Hz EA stimulation at the ST-36 acupoints in STZ-induced diabetic rats, which implies that the cholinergic nerve plays a role. These results are consistent with those reported by Patel [25], who found that the parasympathetic nerves in diabetic rats play an important role in hypoglycemia. Parasympathetic nerves, such as cholinergic nerves secrete Ach. In order to investigate whether the plasma glucose lowering effects of EA are mediated by the cholinergic nerves, we used the acethylcholinesterase (AchE) inhibitor eserine as a cholinergic agonist to induce Ach accumulation in rats prior to EA stimulation. We found that EA stimulation at the ST-36 acupoint 30 min after injection of eserine (0.01 mg kg^−1^ i.p.) resulted in a significant decrease in mean plasma glucose levels. In addition, plasma glucose levels dropped significantly after direct injection of Ach (0.01 mg kg^−1^ i.v.) into the femoral vein, further demonstrating that Ach controls plasma glucose levels. As a drug that indirectly inhibits choline reuptake, HC-3 reduces Ach concentration between synapses [26, 27]. In doing so, HC-3 inhibits cholinergic nerve activity. We found that HC-3 (5 *μ*g kg^−1^ i.p.) blocks the hypoglycemic effects of EA stimulation at ST-36 acupoints in diabetic rats.

In this study, we examined the role adrenal glands play in mediating the hypoglycemic effect of EA stimulation. We found that EA did not result in a significant decrease in mean plasma glucose levels in STZ-adrenalectomized rats. Therefore, our findings indicate that hypoglycemic effect of EA is not only mediated by the cholinergic nerves but also by the adrenal glands (Figure 3). Western blot assay detected the expression of the insulin signaling proteins IRS1 and AKT2 in muscle tissue taken from rats that had been stimulated by EA for 30 min; however, atropine treatment attenuated the expression of those signaling proteins (Figure 2).

Parasympathetic activity mainly effects insulin secretion [28]; however, insulin secretion is impaired in STZ-induced diabetic rats, which leads to high plasma glucose levels. Hyperglycemic stress activates a non-insulin-dependent pathway for glucose uptake in STZ rats. Cholinergic nerve stimulation by EA may play a role in that pathway by triggering the release of proteins involved in the insulin-signaling cascade, such as insulin-like growth factor and *β*-endorphin [13, 29]. Relative studies have also shown the non-insulin-dependent hypoglycemic effect in the *μ*-opioid receptor agonist tramadol [21] and mediation of the protein kinase C (PKC) in *μ*-opioid receptor signaling for glucose uptake in myoblast C_2_C_12_ cells [30]. Whether or not the effects of EA are mediated by the adrenal glands in STZ rats deserves further study.

Our findings indicate that EA (15 Hz) at the ST-36 acupoint induces a hypoglycemic response in STZ-induced diabetic rats by stimulating the cholinergic nerves and involving adrenal glands, which in turn stimulate the expression of insulin signaling proteins.

## Funding

Taichung Veterans General Hospital and DaYeh University, Taiwan (TCVGH-DYU-988310) and China Medical University Hospital, Taiwan (DMR-98-003).

## Figures and Tables

**Table 1:** Influence of atropine, eserine and HC-3 on plasma glucose levels in STZ rats that underwent EA treatment at ST-36.

Group (*n* = 6)	Before	After	HGA%
Atropine + EA	413 ± 29	408 ± 32	–1 ± 4
Atropine	480 ± 51	480 ± 54	0 ± 2
Eserine + EA	406 ± 55	331 ± 57**	–19 ± 6^#^
Eserine	460 ± 111	417 ± 105	–9 ± 6
HC-3 + EA	510 ± 109	494 ± 106	–3 ± 7
HC-3	491 ± 61	471 ± 50	–4 ± 4

The levels of plasma glucose are expressed as mean ± SEM (mg dl^−1^), *n*: sample number; atropine: atropine 0.1 mg kg^−1^ i.p. treatment; atropine + EA: atropine 0.1 mg kg^−1^ i.p. treatment prior to EA; eserine + EA: eserine 0.01 mg kg^−1^ i.p. treatment prior to EA; HC-3: HC-3, 5 *μ*g kg^−1^ i.p. treatment; Before: plasma glucose levels 30 min following medicinal treatment; After: plasma glucose levels following 30 min of EA treatment to ST-36; HGA%: hypoglycemic activity; comparison of before and after EA treatment by self-pair *t*-test **P < .01 versus before; Student's *t*-test ^*#*^ *P* < .05 versus the control group.

**Table 2:** Hypoglycemic response to EA in STZ-adrenalectomized rats.

Group (*n* = 6)	Before	After	HGA%
ADX-EA	507 ± 39	494 ± 63	–3 ± 6
ADX-non EA	460 ± 75	446 ± 78	–3 ± 3

The level of plasma glucose are expressed as mean ± SEM (mg dL^−1^), *n*: sample number; ADX-non-EA: adrenal glands were removed + STZ induced but did not undergo EA; ADX-EA: adrenal glands were removed + STZ induced, followed by EA; before: before EA; after: after EA treatment at ST-36; comparison of plasma glucose levels before and after EA by self-pair *t*-test statistical analysis.
